# Ergostane-Type Sterols from King Trumpet Mushroom (*Pleurotus eryngii*) and Their Inhibitory Effects on Aromatase

**DOI:** 10.3390/ijms18112479

**Published:** 2017-11-21

**Authors:** Takashi Kikuchi, Naoki Motoyashiki, Takeshi Yamada, Kanae Shibatani, Kiyofumi Ninomiya, Toshio Morikawa, Reiko Tanaka

**Affiliations:** 1 Faculty of Pharmaceutical Sciences, Osaka University of Pharmaceutical Sciences, 4-20-1 Nasahara, Takatsuki, Osaka 569-1094, Japan; t.kikuchi@gly.oups.ac.jp (T.K.); mottchan050321@gmail.com (N.M.); yamada@gly.oups.ac.jp (T.Y.); 2 Pharmaceutical Research and Technology Institute, Kindai University, 3-4-1 Kowakae, Higashi-osaka, Osaka 577-8502, Japan; 1633420001c@kindai.ac.jp (K.S.); ninomiya@phar.kindai.ac.jp (K.N.)

**Keywords:** *Pleurotus eryngii*, sterol, ergostane, aromatase inhibitor

## Abstract

Two new ergostane-type sterols; (22*E*)-5*α*,6α-epoxyergosta-8,14,22-triene-3β,7β-diol (**1**) and 5α,6α-epoxyergost-8(14)-ene-3β,7α-diol (**2**) were isolated from the fruiting bodies of king trumpet mushroom (*Pleurotus eryngii*), along with eight known compounds (**3**–**10**). All isolated compounds were evaluated for their inhibitory effects on aromatase. Among them, **4** and **6** exhibited comparable aromatase inhibitory activities to aminoglutethimide.

## 1. Introduction

Estrogen is responsible for breast cancer growth. The target genes of an estrogen receptor are in control of cancer cell development in estrogen-dependent breast tumors. Binding of estrogen receptor to estrogen triggers transcription of its target genes [1]. Aromatase is the rate-limiting enzyme in estrogen biosynthesis [1]. This enzyme converts androgens (testosterone and androtestosterone) into estrogens (estradiol and estrone, respectively) [2]. Aromatase inhibitors (AIs) are adjuvant in hormone treatments commonly prescribed for breast cancers that are hormone receptor-positive in the early stage [3]. However, the currently used AIs have several side effects of menopausal symptoms such as hot flashes, vaginal dryness, sexual dysfunction, musculoskeletal symptoms, osteoporosis, bone fracture, fatigue, mood disturbance, nausea, and vomiting [3]. Therefore, natural compounds obtained from safe food resources might be useful in the search for promoter-specific AIs with few side effects [4].

*Pleurotus eryngii* (Japanese name: eringi, English name: oyster mushroom or king trumpet) is an edible mushroom. *P. eryngii* is native to North Africa, Asia, and Europe [5], and also grown commercially in Japan, China, and the US [6]. Previous studies demonstrated the inhibitory effects on human neutrophil elastase (HNE) [7], antioxidant and antimutagenic activities [8], and inhibitory effects on allergic mediators [9] of *P. eryngii* extracts. *P. eryngii* contains amino acids, vitamins, and dietary fiber [10]. It also includes polysaccharides [11,12], pleurone [7], ergostane-type sterols [13], and eryngiolide A [14]. These chemical constituents exhibit biological activities such as the antioxidant [11] and antitumor activities [12] of a polysaccharide, HNE-inhibitory effects of pleurone [7], and cytotoxicity against human cancer cell lines of eryngiolide A [14]. We recently reported eringiacetal A, which is an ergostane-type sterol with a cage-shaped structure [15], and a 9,11-*seco*-ergostane and five ergostane-type sterols [16] from the fruiting bodies of *P. eryngii*. In a continuing study, we isolated 10 ergostane-type sterols, and elucidated the structures of two new compounds; (22*E*)-5α,6α-epoxyergosta-8,14,22-triene-3β,7β-diol (**1**), and 5α,6α-epoxyergost-8(14)-ene-3β,7α-diol (**2**). In addition, the isolated constituents were evaluated for inhibitory activities on aromatase.

## 2. Results

### 2.1. Isolation and Structure Elucidation

**Figure 1:**
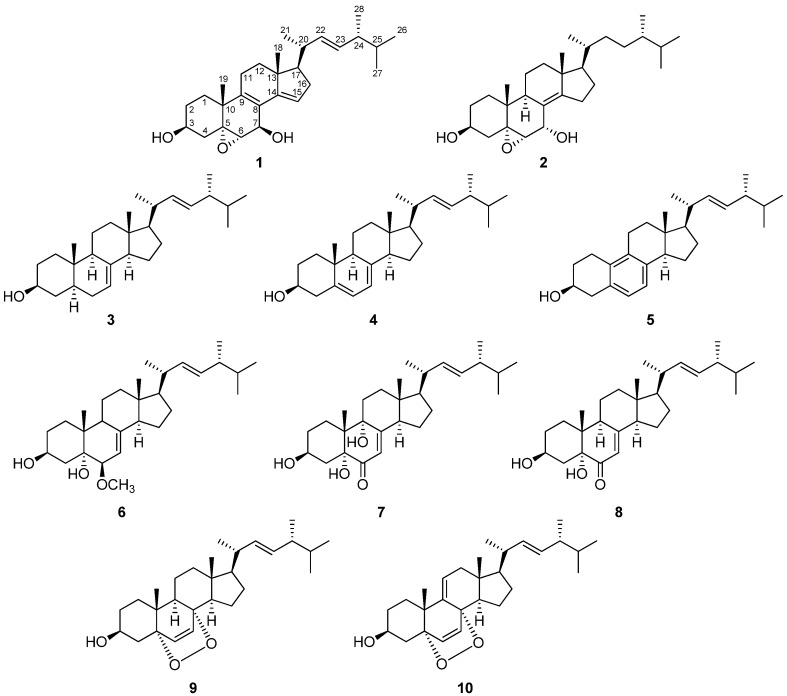
Structures of compounds.

(22*E*)-Ergosta-7,22-dien-3β-ol (**3**) [17], (22*E*)-ergosta-5,7,22-trien-3β-ol (**4**) [18], (22*E*)-19-norergosta-5,7,9,22-tetraen-3β-ol (**5**) [19], ergosterol peroxide (**9**) [18], and 9,11-dehydroergosterol peroxide (**10**) [20] were isolated from sample 1, and Compounds **1**, **2**, (22*E*)-6β-methoxyergosta-7,22-diene-3β,5α-diol (**6**) [21], (22*E*)-3β,5α,9α-trihydroxyergosta-7,22-dien-6-one (**7**) [21], and (22*E*)-3β,5α-dihydroxyergosta-7,22-dien-6-one (**8**) [22] were obtained from sample 2 (Figure 1). Of these, **1** and **2** were new compounds.

**Figure 2:**
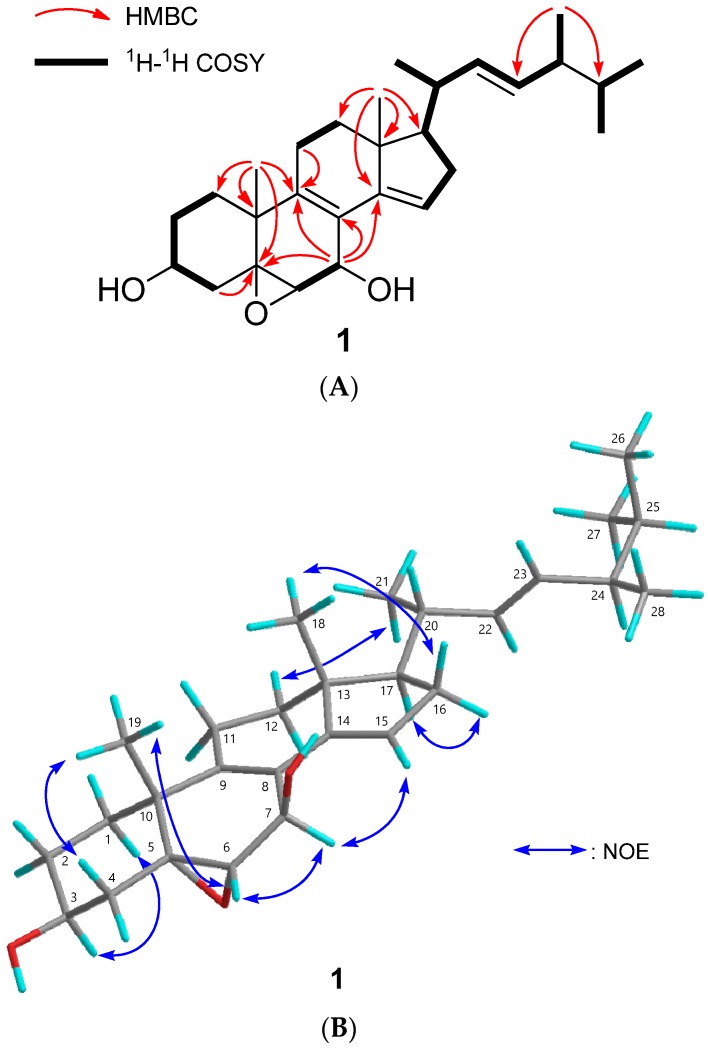
Structure determination of compound **1**. (**A**) Key HMBC and ^1^H-^1^H COSY correlations of compound **1**; (**B**) Key NOE correlations of compound **1.** The atoms of C, H, and O were shown in grey, aqua, and red, respectively.

Compound **1** was isolated as an amorphous solid, with a molecular formula of C_28_H_42_O_3_ by HREIMS. The infrared (IR) spectrum indicated the presence of hydroxy groups (*ν*_max_ 3451 cm^−1^), and the UV spectrum suggested the presence of a conjugated diene (*λ*_max_ 242.0 nm). The ^1^H and ^13^C NMR spectra (*δ*_H_ and *δ*_C_ in ppm) in CDCl_3_ displayed signals for two tertiary methyls (*δ*_H_ 0.82 (singlet (s)), 1.30 (s)), four secondary methyls (*δ*_H_ 0.83 (doublet (d)), 0.85 (d), 0.93 (d), 1.04 (d)), three oxymethines (*δ*_H_ 3.24 (d), 3.96 (triplet of triplets (tt)), 4.85 (broad singlet (br s)); *δ*_C_ 59.5 (d), 63.8 (d), 68.4 (d)), an sp^3^ oxygenated quaternary carbon (*δ*_C_ 63.3 (s)), a tetrasubstituted olefin (*δ*_C_ 122.2 (s), 138.8 (s)), a trisubstituted olefin (*δ*_H_ 5.55 (br s); *δ*_C_ 118.7 (d), 147.7 (s)), and a disubstutited olefin (*δ*_H_ 5.20 (doublet of doublets (dd)), 5.28 (dd); *δ*_C_ 132.4 (d), 135.1 (d)) (Table 1, Figures S1–S4). In the HMBC spectrum, the correlations were observed as follows; Me-19 (*δ*_H_ 0.82 (s))/C-5 (*δ*_C_ 63.3 (s)), C-9 (*δ*_C_ 138.8 (s)); H-7 (*δ*_H_ 4.85 (br s))/C-5 (*δ*_C_ 63.3 (s)), C-9 (*δ*_C_ 138.8 (s)), C-14 (*δ*_C_ 147.7 (s)); H-6 (*δ*_H_ 3.24 (d))/C-7 (*δ*_C_ 63.8 (d)), C-8 (*δ*_C_ 122.2 (s)); Me-18 (*δ*_H_ 0.82 (s))/C-14 (*δ*_C_ 147.7 (s)); Me-28 (*δ*_H_ 0.93 (d))/C-23 and C-25 (Figure 2A and Figure S5). The correlations between H_2_-1–H_2_-2–H-3 (*δ*_H_ 3.96 (tt))–H_2_-4; H-6 (*δ*_H_ 3.24 (d))–H-7 (*δ*_H_ 4.85 (br s)); H-15 (*δ*_H_ 5.55 (br s))–H_2_-16–H-17–H-20–Me-21; H-20–H-22 (*δ*_H_ 5.20 (dd))–H-23 (*δ*_H_ 5.28 (dd))–H-24–Me-28 (*δ*_H_ 0.93 (d)); Me-26 (*δ*_H_ 0.85 (d))–H-25–Me-27 (*δ*_H_ 0.83 (d)) were observed in the ^1^H-^1^H COSY spectrum (Figure 2A and Figure S6). From the above, the planar structure was determined as shown in Figure 2A. The configuration of the hydroxy groups at the C-3 position was determined as β-orientation because of the coupling constant (*J*) (*δ*_H_ 3.96 (tt, 11.5, 5.4 Hz)). The NOE correlation of Me-19/H-6β (equatrial) suggested that the epoxy group at C-5,6 was α-oriented, and that of H-7α/H-15 suggested that 7-OH was β-oriented (Figure 2B and Figure S7). The geometry of the double bond at C-22 was determined as *E* from the coupling constants of H-22 (*δ*_H_ 5.20 (dd, *J* = 15.2, 7.6 Hz)) and H-23 (*δ*_H_ 5.28 (dd, *J* = 15.2, 7.9 Hz)). Comparison of ^13^C NMR chemical shifts at C-24 (*δ*_C_ 42.8) and 28 (*δ*_C_ 17.6) with those of 24*R* (*δ*_C_ 42.9 (C-24) and 17.7 (C-28)) and 24*S* (*δ*_C_ 43.2 (C-24) and 18.1 (C-28)) methylcholestane-type sterols [23,24] established the stereochemistry of C-24 as *R*. Therefore compound **1** was determined as (22*E*)-5α,6α-epoxyergosta-8,14,22-triene-3β,7β-diol (Figure 1, Table S1). Compound **1** was similar to (22*E*)-5α,6α-epoxy-ergosta-8,14,22-triene-7β,7α-diol [25], except for the absence of a 7α-hydroxy group and the presence of a 7β-hydroxy group. There are differences in *δ*_H_ value measured with C_6_D_6_ such as H-7 (7α-hydroxy-type: *δ*_H_ 4.34 (1H, dd, *J* = 11.2, 2.6 Hz) [25] vs. 7β-hydroxy-type (**1**): *δ*_H_ 4.74 (br s)), and H-15 (7α-hydroxy-type: *δ*_H_ 6.50 (1H, dd, *J* = 3.3, 1.8 Hz) [25] vs. 7β-hydroxy-type (**1**): *δ*_H_ 5.33 (br s)).

**Figure 3:**
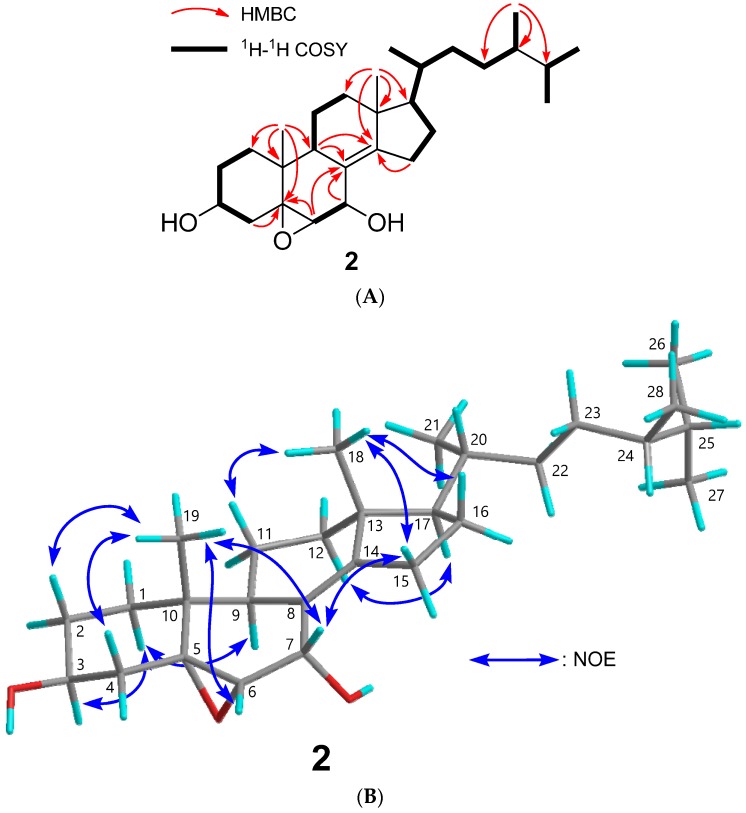
Structure determination of compound **2**. (**A**) Key HMBC and ^1^H-^1^H COSY correlations of compound **2**; (**B**) Key NOE correlations of compound **2**. The atoms of C, H, and O were shown in grey, aqua, and red, respectively.

Compound **2** was isolated as an amorphous solid, with a molecular formula of C_28_H_46_O_3_. The IR spectrum suggested the presence of hydroxy groups (3387 cm^−1^). The ^1^H, ^13^C NMR and HSQC spectra indicated the presence of two tertiary methyls (*δ*_H_ 0.85 (s), 0.87 (s)), four secondary methyls (*δ*_H_ 0.77 (d), 0.78 (d), 0.85 (d), 0.93 (d)), two oxymethines (*δ*_H_ 3.92 (tt), 4.43 (dd); *δ*_C_ 65.1 (d), 68.7 (d)), a trisubstituted epoxy (*δ*_H_ 3.15 (d); *δ*_C_ 61.3 (d), 67.8 (s)), and a tetrasubstituted olefin (*δ*_C_ 125.1 (s), 152.7 (s)) (Table 1, Figures S8–S11). Based on the correlations at Me-18/C-14 (*δ*_C_ 152.7 (s)), Me-19/C-5 (*δ*_C_ 67.8 (s)), and H-15/C-8 (*δ*_C_ 125.1 (s)) and C-14 (*δ*_C_ 152.7 (s)) in the HMBC spectrum, and H_2_-1–H_2_-2–H-3 (*δ*_H_ 3.92 (tt))–H_2_-4; H-6 (*δ*_H_ 3.15 (d))–H-7 (*δ*_H_ 4.43 (dd)) in the ^1^H-^1^H COSY spectrum (Figure 3A, Figures S12 and S13), oxymethines were at C-3 and C-7 positions, a trisubstituted epoxy group at the C-5, 6 positions, and a tetrasubstituted olefin at the C-8, 14 positions (Figure 3A). The NOE correlation between H-7 and Me-19 demonstrated the configuration of the hydroxy group at the C-7 position as α-orientation (Figure 3B and Figure S14). The NOE correlation between H-4β and Me-19 suggested the orientation of the epoxy group at C-5, 6 was α (Figure 3B and Figure S14). The stereochemistry of C-24 was established as *S* by comparison of the ^1^H NMR chemical shift at Me-28 (*δ*_H_ 0.77) with those of 24*R* (*δ*_H_ 0.802) and 24*S* (*δ*_H_ 0.781) ergostane-type sterols [26,27]. Therefore, the structure of **2** was established as 5α,6α-epoxyergosta-8(14)-ene-3β,7α-diol (Figure 1, Table S2).

### 2.2. Evaluation for Aromatase Inhibitory Effects

**Figure 4:**
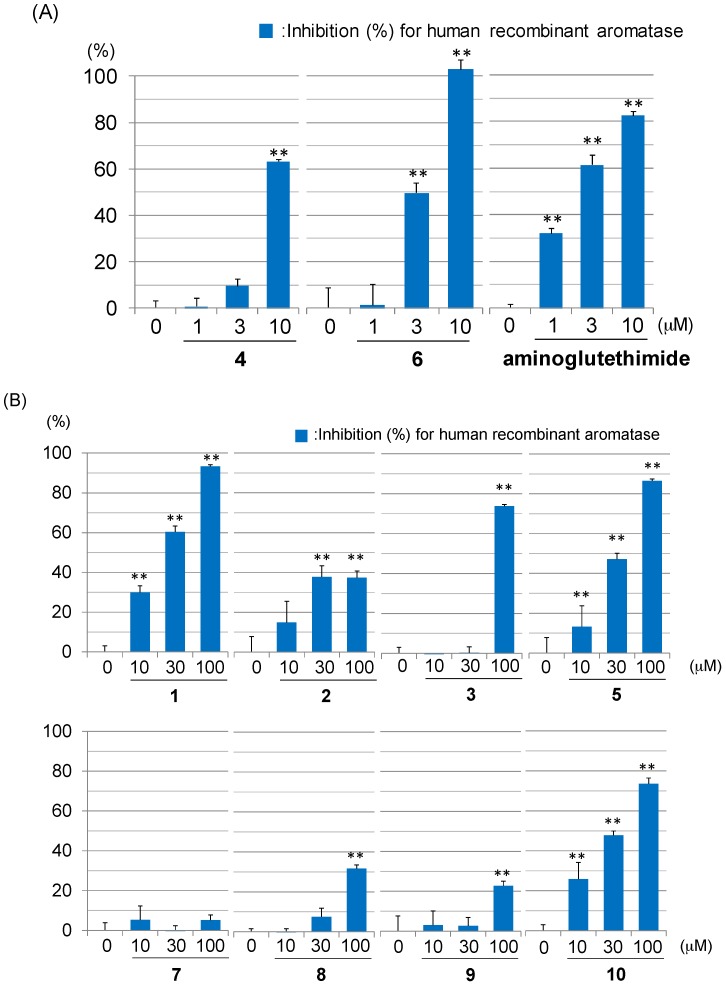
Inhibitory effects of sterols (**1**–**10**) from *P. eryngii* against human recombinant aromatase. (**A**) Inhibitory effects of sterols (**4**, **6**) and aminoglutethimide at 1, 3, and 10 µM. (**B**) Inhibitory effects of sterols (**1–3**, **5**, **7**–**10**) at 10, 30, and 100 µM. Each value represents the mean ± the standard error (S.E.) of three determinations. Significant differences from the vehicle control (0 μM) group shown as ** *p* < 0.01.

Compounds **1**–**10** and aminoglutethimide, a positive control, were evaluated for their aromatase inhibitory activities. Compounds **4** and **6** exhibited comparable inhibitory activities (IC_50_ **4**: 8.1 µM; **6**: 2.8 µM) to aminoglutethimide (IC_50_ 2.0 µM) (Figure 4A). Compounds **1**, **3**, **5**, and **10** showed moderate activities (IC_50_ **1**: 17.3 µM; **3**: 66.1 µM; **5**: 33.8 µM; **10**: 32.6 µM) (Figure 4B). Compounds **2**, **7**, **8**, and **9** weakly inhibited aromatase (Figure 4B). Above results suggested that compounds **4** and **6** can be regarded as potential anti-breast cancer agents targeting aromatase. Based on the results in the figures, the following structure-activity relationship of the compounds can be concluded: (i) The double-bond at C-5, 6 intensifies the aromatase inhibitory activity in ergost-7-ene compounds (**3** (IC_50_ 66.1 µM) vs. **4** (IC_50_ 8.1 µM)); (ii) 9(11)-double-bond enhances the inhibitory activity in 5α,8α-epidioxyergost-6-ene compounds (**9** (IC_50_ > 100 µM) vs. **10** (IC_50_ 32.6 µM)); (iii) 7-ene-6-one compounds did not show this activity (**7** and **8** (IC_50_ > 100 µM)).

## 3. Experimental Section 

### 3.1. General Methods

Dibenzylfluorescein (DBF) and Human CYP19 + P450 Reductase SUPERSOMES (human recombinant aromatase) were obtained from BD Biosciences (Heidelberg, Germany). The physical data were obtained by the following instruments: a Yanagimoto micro-melting point apparatus for melting points (uncorrected); a JASCO DIP-1000 digital polarimeter for Optical rotations; a Perkin-Elmer 1720X FTIR spectrophotometer for IR spectra; an Agilent-NMR-vnmrs600 for the ^1^H and ^13^C NMR spectra (^1^H: 600 MHz; ^13^C: 150 MHz) in CDCl_3_ with tetramethylsilane as the internal standard; a Hitachi M-4000H double-focusing mass spectrometer for EIMS (70 eV). Column chromatography was carried out by Silica gel (70–230 mesh, Merck, Darmstadt, Germany) and silica gel 60 (230–400 mesh, Nacalai Tesque, Inc*.,* Kyoto, Japan). HPLC was performed by the following systems; system I: *Cosmosil 5SL-II column* (25 cm × 20 mm i.d.) (Nacalai Tesque, Inc.), hexane/EtOAc (5:1), 8.0 mL/min, 35 °C; system II: *Shimpack PREP-ODS* (25 cm × 20 mm i.d.) (Shimadzu corp., Kyoto, Japan), MeOH, 8.0 mL/min, 35 °C; system III: *Cosmosil 5C_18_-MS-II column* (25 cm × 20 mm i.d.) (Nacalai Tesque, Inc.), MeOH/H_2_O (95:5), flow rate, 4.0 mL/min, 35 °C; system IV: *Cosmosil 5C_18_-MS-II column*, MeOH/H_2_O (9:1), 4.0 mL/min, 35 °C.

### 3.2. Materials

The fruiting bodies of *P. eryngii* were purchased from HOKUTO Corp. They were cultivated in Kagawa, Japan (Sample 1 in 2011, and Sample 2 in 2014). A voucher material has been deposited in the Herbarium of the Laboratory of Medicinal Chemistry, Osaka University of Pharmaceutical Sciences.

### 3.3. Extraction and Isolation

#### 3.3.1. Sample 1

Sample 1 (fruiting bodies of *P. eryngii* (21 kg, fresh weight)) was extracted with MeOH under reflux (1 week, 4 times). The MeOH extract (170 g) was then divided into EtOAc and H_2_O fractions by liquid-liquid partition. The EtOAc fraction (60 g) was separated into 20 fractions (Fr. *S1*-A to *S1*-T) with SiO_2_ column chromatography (CC) (SiO_2_ (3.5 kg); CHCl_3_/EtOAc (1:0 to 0:1), and EtOAc/MeOH (5:1, and 0:1)).

Fr. *S1*-H (836.5 mg), CHCl_3_/EtOAc (10:1)-eluted fraction, was separated with SiO_2_ CC to yield 8 fractions, *S1*-H1 to *S1*-H8. Preparative HPLC (system I) of *S1*-H3 (185.7 mg), hexane/EtOAc (5:1)-eluted fraction, provided 7 fractions, *S1*-H3-1 to *SF*3-7. *S1*-H3-4 was identified as **4** (31.8 mg; retention time (*t*_R_) 19.2 min). Preparative HPLC (system II) of *S1-*H3-5 (5.4 mg, *t*_R_ 36.5 min) provided **3** (1.9 mg; *t*_R_ 37.5 min). Preparative HPLC (system IV) of *S1-*H6 (14.3 mg), hexane/EtOAc (3:1)-eluted fraction, provided **10** (1.3 mg, *t*_R_ 95.4 min) and **9** (1.7 mg, *t*_R_ 120.2 min).

Fr. *S1-*I (1072.3 mg), CHCl_3_/EtOAc (10:1)-eluted fraction, was separated with SiO_2_ CC to give 8 fractions, *S1*-I1 to *S1*-I10. Preparative HPLC (system I) of *S1*-I5 (45.2 mg), hexane/EtOAc (5:1)-eluted fraction, provided **5** (2.8 mg, *t*_R_ 42.7 min).

#### 3.3.2. Sample 2

Sample 2 (fruiting bodies of *P. eryngii* (120 kg, fresh weight)) was extracted with MeOH under reflux (3 days, 4 times). The MeOH extract (2625 g) was divided into EtOAc and H_2_O fractions by liquid-liquid partition. The EtOAc fraction (240 g) was separated into 37 fractions (Fr. *S2*-A to *S2*-Z, and *S2*-a to *S2*-k) with SiO_2_ column chromatography (CC) (SiO_2_ (2.8 kg); CHCl_3_/EtOAc (1:0 to 0:1), and MeOH).

Fr. *S2-*V (3964.9 mg), CHCl_3_/EtOAc (1:1)-eluted fraction, was separated by SiO_2_ CC to give 8 fractions, *S2-*V1 to *S2-*V21. Preparative HPLC (system III) of Fr. *S2-*V4 (110.9 mg), hexane/EtOAc (1:1)-eluted fraction, provided **8** (1.5 mg; *t*_R_ 36.9 min) and **6** (6.3 mg; *t*_R_ 49.5 min). Preparative HPLC (system IV) of Fr. *S2*-V6 (451.6 mg), hexane/EtOAc (1:1)-eluted fraction, provided **7** (1.1 mg; *t*_R_ 59.8 min). Preparative HPLC (system IV) of Fr. *S2*-V7 (270.5 mg), hexane/EtOAc (1:1)-eluted fraction, provided **2** (3.0 mg; *t*_R_ 76.8 min). Preparative HPLC (system III) of Fr. *S2*-V10 (270.5 mg), hexane/EtOAc (1:1)-eluted fraction, provided **1** (1.9 mg; *t*_R_ 36.3 min).

#### 3.3.3. (22*E*)-5α,6α-Epoxyergosta-8,14,22-triene-3β,7β-diol (**1**) 

[α]^20^_D_ −23.6 (*c* = 0.13, EtOH); IR *ν*_max_^KBr^ cm^−1^: 3451, 2960, 1697, 1557, 1456; UV *λ*_max_^EtO^^H^ nm (log*ε*): 206.0 (3.75), 242.0 (3.62); EIMS *m*/*z*: 426 [M]^+^ (71), 315 (27), 300 (57), 172 (83), 69 (100); HREIMS *m*/*z*: 426.3127 [M]^+^ (calcd for 426.3134: C_28_H_42_O_3_); ^1^H NMR (400 MHz, C_6_D_6_) *δ*_H_ ppm: 0.80 (s, H-19), 0.91 (d, 6.8 Hz), 0.93 (d, 6.4 Hz), 1.01 (d, 6.8 Hz), 1.07 (d, 6.4 Hz), 1.12 (s, H-18), 3.11 (d, 2.4 Hz, H-6), 3.83 (tt, 11.6, 4.8 Hz, H-3), 4.74 (br s, H-7), 5.24 (dd, 15.6, 8.4 Hz, H-22), 5.32 (overlapped, H-23), 5.33 (br s, H-15).

#### 3.3.4. 5α,6α-Epoxyergost-8(14)-ene-3β,7α-diol (**2**)

[α]^20^_D_ −112.2 (*c* = 0.13, EtOH); IR *ν*_max_^KBr^ cm^−1^: 3387, 2959, 2936, 2871, 1466, 1377; EIMS *m*/*z*: 430 [M]^+^ (5), 412 (100), 394 (57), 379 (58), 267 (23), 213 (21); HREIMS *m*/*z*: 430.3450 [M]^+^ (calcd for 430.3447: C_28_H_46_O_3_).

### 3.4. Inhibitory Effects against Human Recombinant Aromatase

Inhibitory assay against human recombinant aromatase was performed as described previously [28,29]. 

### 3.5. Statistics

Values are described as the mean ± standard error of the mean (S.E.M.). Statistical analysis was performed by one-way analysis of variance, followed by Dunnett’s test. Probability (*p*) values less than 0.05 were regarded as significant.

## 4. Conclusions

In this study, we isolated two new sterols (**1** and **2**) and elucidated their structures. They have 5α,6α-epoxy-7-hydroxy ergostane structure. In aromatase inhibitory assay, compounds **4** and **6** possessed comparable inhibitory effects (IC_50_ **4**: 8.1 µM; **6**: 2.8 µM) against human recombinant aromatase to aminoglutethimide (IC_50_ 2.0 µM). These results suggested that compounds **4** and **6** have potential as anti-breast cancer agents.

## Figures and Tables

**Table 1:** ^1^H and ^13^C NMR Data for Compounds **1** and **2** in CDCl_3_ (*δ* in ppm; *J* in Hz).

	1				2			
Position	***δ*_H_**		***δ*_C_**		***δ*_H_**		***δ*_C_**	
1α	2.01	(1H, multiplet (m))	31.0	t	1.46	(1H, m)	32.2	t
1β	1.86	(1H, m)			1.67	(1H, m)		
2	1.68	(2H, m)	30.9	t	α 1.96	(1H, m)	31.1	t
					β 1.56	(1H, m)		
3	3.96	(1H, tt, *J* = 11.5, 5.4)	68.4	d	3.92	(1H, tt, *J* = 11.4, 3.0)	68.7	d
4α	1.50	(1H, m)	39.0	t	1.42	(1H, m)	39.6	t
4β	2.21	(1H, m)			2.13	(1H, dd, *J* = 13.2, 11.4)		
5			63.3	s			67.8	s
6	3.24	(1H, d, *J* = 2.4)	59.5	d	3.15	(1H, d, *J* = 3.5)	61.3	d
7	4.85	(1H, br s)	63.8	d	4.43	(1H, dd, *J* = 9.6, 3.5)	65.1	d
8			122.2	s			125.1	s
9			138.8	s	2.35	(1H, m)	38.7	d
10			38.3	s			35.8	s
11	2.19	(2H, m)	22.2	t	α 1.49	(1H, m)	19.0	t
					β 1.40	(1H, m)		
12α	1.47	(1H, m)	35.4	t	1.16	(1H, m)	36.7	t
12β	1.99	(1H, m)			1.95	(1H, m)		
13			44.6	s			43.1	s
14			147.7	s			152.7	s
15	5.55	(1H, br s)	118.7	d	α 2.65	(1H, m)	25.0	t
					β 2.30	(1H, m)		
16α	2.27	(1H, m)			1.89	(1H, m)	26.6	t
16β	2.08	(1H, m)	36.8	t	1.41	(1H, m)		
17	1.55	(1H, m)	56.4	d	1.21	(1H, m)	56.6	d
18	0.82	(3H, s)	15.6	quartet (q)	0.85	(3H, s)	17.9	q
19	1.30	(3H, s)	23.6	q	0.87	(3H, s)	16.5	q
20	2.24	(1H, m)	38.8	d	1.46	(1H, m)	34.9	d
21	1.04	(3H, d, *J* = 6.5)	21.0	q	0.93	(3H, d, *J* = 6.8)	19.1	q
22	5.20	(1H, dd, *J* = 15.2, 7.6)	135.1	d	A 1.03	(1H, m)	33.4	t
					B 1.44	(1H, m)		
23	5.28	(1H, dd, *J* = 15.2, 7.9)	132.4	d	A 0.95	(1H, m)	30.4	t
					B 1.37	(1H, m)		
24	1.88	(1H, m)	42.8	d	1.21	(1H, m)	39.1	d
25	1.48	(1H, m)	33.1	d	1.58	(1H, m)	31.5	d
26	0.85	(3H, d, *J* = 6.8)	19.9	q	0.85	(3H, d, *J* = 7.1)	20.5	q
27	0.83	(3H, d, *J* = 6.8)	19.6	q	0.78	(3H, d, *J* = 7.0)	17.6	q
28	0.93	(3H, d, *J* = 6.8)	17.6	q	0.77	(3H, d, *J* = 6.9)	15.4	q

## Supplementary Materials

Supplementary materials can be found at www.mdpi.com/1422-0067/18/11/2479/s1.

## Abbreviations

NMR	Nuclear magnetic resonance
HREIMS	High resolution electron ionization mass spectrometry
CDCl_3_	Duterated chloroform
HMBC	Heteronuclear multiple bond coherence
COSY	Correlation spectroscopy
NOE	Nuclear overhauser effect
HSQC	Hetero nuclear single quantum coherence
