# Supplementary material for: Ergostane-Type Sterols from King Trumpet Mushroom (Pleurotus eryngii) and Their Inhibitory Effects on Aromatase
Source: Int J Mol Sci. 2017 Nov 21;18(11):2479. doi: 10.3390/ijms18112479 (PMC5713445; doi:10.3390/ijms18112479)

Supporting information

Ergostane-Type Sterols from King Trumpet Mushroom (*Pleurotus eryngii*) and Their Inhibitory Effects on Aromatase

Takashi Kikuchi[1], Naoki Motoyashiki[1], Takeshi Yamada[1], Kanae Shibatani[2], Kiyofumi Ninomiya[2], Toshio Morikawa[2,*], Reiko Tanaka[1,*]

[1] Osaka University of Pharmaceutical Sciences, 4-20-1 Nasahara, Takatsuki, Osaka 569-1094, Japan
[2] Pharmaceutical Research and Technology Institute, Kindai University, 3-4-1 Kowakae, Higashi-osaka,Osaka 577-8502, Japan

* Correspondence: tanakar@gly.oups.ac.jp (R.T.); morikawa@kindai.ac.jp (T.M.);
Tel.: +81-72-690-1084 (R.T.); +81-6-4307-4306 (T.M.); Fax: +81-72-690-1084 (R.T.); +81-6-6729-3577 (T.M.)

Supporting Information

Table S1. NMR Data for Compound **1**

Table S2. NMR Data for Compound **2**

Figure S1. ^{1}H NMR spectrum of compound **1**.

Figure S2. ^{13}C NMR spectrum of compound **1**.

Figure S3. DEPT spectrum of compound **1**.

Figure S4. HSQC spectrum of compound **1**.

Figure S5. HMBC spectrum of compound **1**.

Figure S6. ^{1}H-^{1}H COSY spectrum of compound **1**.

Figure S7. NOESY spectrum of compound **1**.

Figure S8. ^{1}H NMR spectrum of compound **2**.

Figure S9. ^{13}C NMR spectrum of compound **2**.

Figure S10. DEPT spectrum of compound **2**.

Figure S11. HSQC spectrum of compound **2**.

Figure S12. HMBC spectrum of compound **2**.

Figure S13. ^{1}H-^{1}H COSY spectrum of compound **2**.

Figure S14. NOESY spectrum of compound **2**.

Table S1. NMR Data for Compound 1 in $CDCl_3$ (δ in ppm; J in Hz)

	δ_H		^{1}H-^{1}H COSY	NOE	δ_C		HMBC (H to C)
1α	2.01	(1H, m)	1β, 2	3	31.0	t	2, 3, 19
1β	1.86	(1H, m)	1α, 2				
2	1.68	(2H, m)	1α, 1β, 3	19	30.9	t	
3	3.96	(1H, tt, J = 11.5, 5.4)	2, 4α, 4β	1α	68.4	d	
4α	1.50	(1H, m)	3	6	39.0	t	3, 5
4β	2.21	(1H, m)	3	19			3
5					63.3	s	
6	3.24	(1H, d, J = 2.4)	7	4α, 7	59.5	d	7, 8
7	4.85	(1H, br s)		6, 15	63.8	d	5, 6, 8, 14
8					122.2	s	
9					138.8	s	
10					38.3	s	
11	2.19	(2H, m)	12α, 12β		22.2	t	9
12α	1.47	(1H, m)	11		35.4	t	
12β	1.99	(1H, m)	11	21			
13					44.6	s	
14					147.7	s	
15	5.55	(1H, br s)	16α, 16β	7	118.7	d	13, 16, 17
16α	2.27	(1H, m)	15, 17	17			13, 14, 15, 17
16β	2.08	(1H, m)	15, 17	18	36.8	t	14, 15, 17
17	1.55	(1H, m)	16, 20		56.4	d	12, 13, 16
18	0.82	(3H, s)		16β, 20	15.6	q	12, 13, 14, 17
19	1.30	(3H, s)		2β, 4β	23.6	q	1, 5, 9, 10
20	2.24	(1H, m)	17, 21, 22	18	38.8	d	22, 23
21	1.04	(3H, d, J = 6.5)	20	12β	21.0	q	17, 20, 22
22	5.20	(1H, dd, J = 15.2, 7.6)	20, 23		135.1	d	21, 23, 24
23	5.28	(1H, dd, J = 15.2, 7.9)	22		132.4	d	22, 24, 28
24	1.88	(1H, m)	23, 28		42.8	d	22, 23, 25, 26
25	1.48	(1H, m)	26, 27		33.1	d	23, 24, 26, 28
26	0.85	(3H, d, J = 6.8)	25		19.9	q	24, 25, 27
27	0.83	(3H, d, J = 6.8)	25		19.6	q	24, 25, 26
28	0.93	(3H, d, J = 6.8)	24		17.6	q	23, 24, 25

Table S2. NMR Data for Compound 2 in $CDCl_3$ (δ in ppm; J in Hz)

	δ_H	^1H-^1H COSY	NOE	δ_C		HMBC (H to C)
1α	1.46 (1H, m)	1β, 2α, 2β	3	32.2	t	2, 3, 5, 9, 10
1β	1.67 (1H, m)	1α, 2α, 2β				2, 3, 5, 10
2α	1.96 (1H, m)	1α, 1β, 2β, 3		31.1	t	9
2β	1.56 (1H, m)	1α, 1β, 2α, 3	19			1
3	3.92 (1H, tt, J = 11.4, 3.0)	2α, 2β, 4α, 4β	1α	68.7	d	
4α	1.42 (1H, m)	3, 4β	6	39.6	t	2, 3, 5, 6, 10
4β	2.13 (1H, dd, J = 13.2, 11.4)	3, 4α	19			2, 3
5				67.8	s	
6	3.15 (1H, d, J = 3.5)	7	4α, 19	61.3	d	4, 5, 7, 8
7	4.43 (1H, dd, J = 9.6, 3.5)	6	19	65.1	d	8
8				125.1	s	
9	2.35 (1H, m)	11α, 11β		38.7	d	8, 10, 11, 14, 19
10				35.8	s	
11α	1.49 (1H, m)	9, 11β		19.0	t	8, 13
11β	1.40 (1H, m)	9, 11α	18			9, 10, 13
12α	1.16 (1H, m)	11α, 11β, 12β	17	36.7	t	18
12β	1.95 (1H, m)	11α, 11β, 12α				13, 14, 18
13				43.1	s	
14				152.7	s	
15α	2.65 (1H, m)	15β, 16α, 16β		25.0	t	8, 13, 14, 16
15β	2.30 (1H, m)	15α, 16β	18			8, 10, 14, 16, 17
16α	1.89 (1H, m)	15α, 16β		26.6	t	13, 14, 15, 17
16β	1.41 (1H, m)	15α, 15β, 16α	18			13, 15, 17, 20
17	1.21 (1H, m)	16α, 16β, 20	12α	56.6	d	12, 13, 20, 22
18	0.85 (3H, s)		11β, 15β, 16β	17.9	q	12, 13, 14, 17
19	0.87 (3H, s)		2β, 4β, 6, 7	16.5	q	1, 5, 9, 10
20	1.46 (1H, m)	17, 21, 22A, 22B		34.9	d	13, 17
21	0.93 (3H, d, J = 6.8)	20		19.1	q	17, 20, 22
22A	1.03 (1H, m)	20, 22B, 23A, 23B		33.4	t	20, 21, 23
22B	1.44 (1H, m)	20, 22A, 23A, 23B				20, 24
23A	0.95 (1H, m)	22A, 22B, 23B, 24		30.4	t	
23B	1.37 (1H, m)	22A, 22B, 23A, 24				22, 24, 25, 28
24	1.21 (1H, m)	26		39.1	d	22, 23, 24, 26, 28
25	1.58 (1H, m)	23A, 23B, 28		31.5	d	23, 25, 26, 28
26	0.85 (3H, d, J = 7.1)	24		20.5	q	24, 27
27	0.78 (3H, d, J = 7.0)	25		17.6	q	24, 26
28	0.77 (3H, d, J = 6.9)	25		15.4	q	23, 24, 25

Figure S1. ^{1}H NMR spectrum of compound **1**.

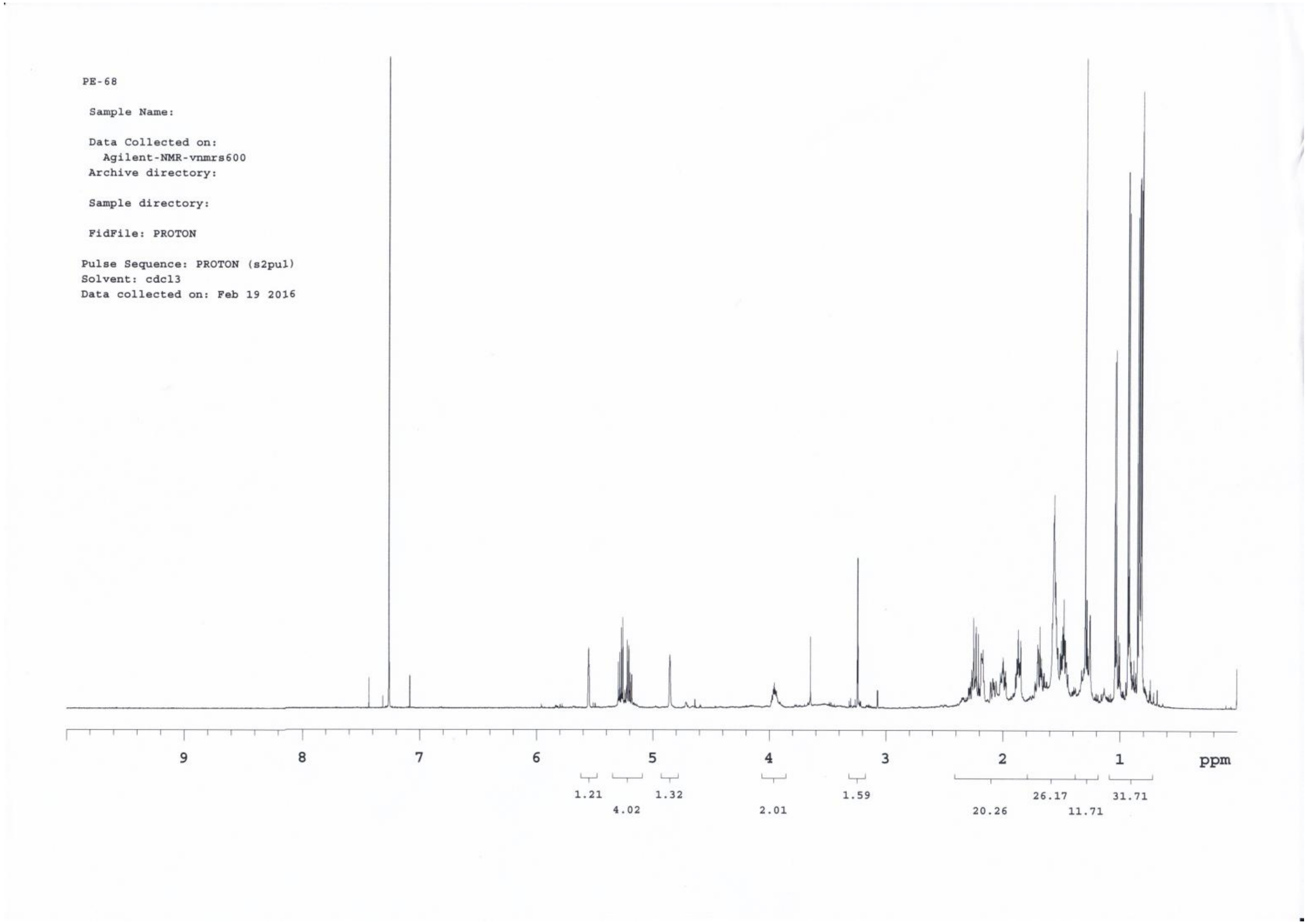

Figure S2. ^{13}C NMR spectrum of compound **1**.

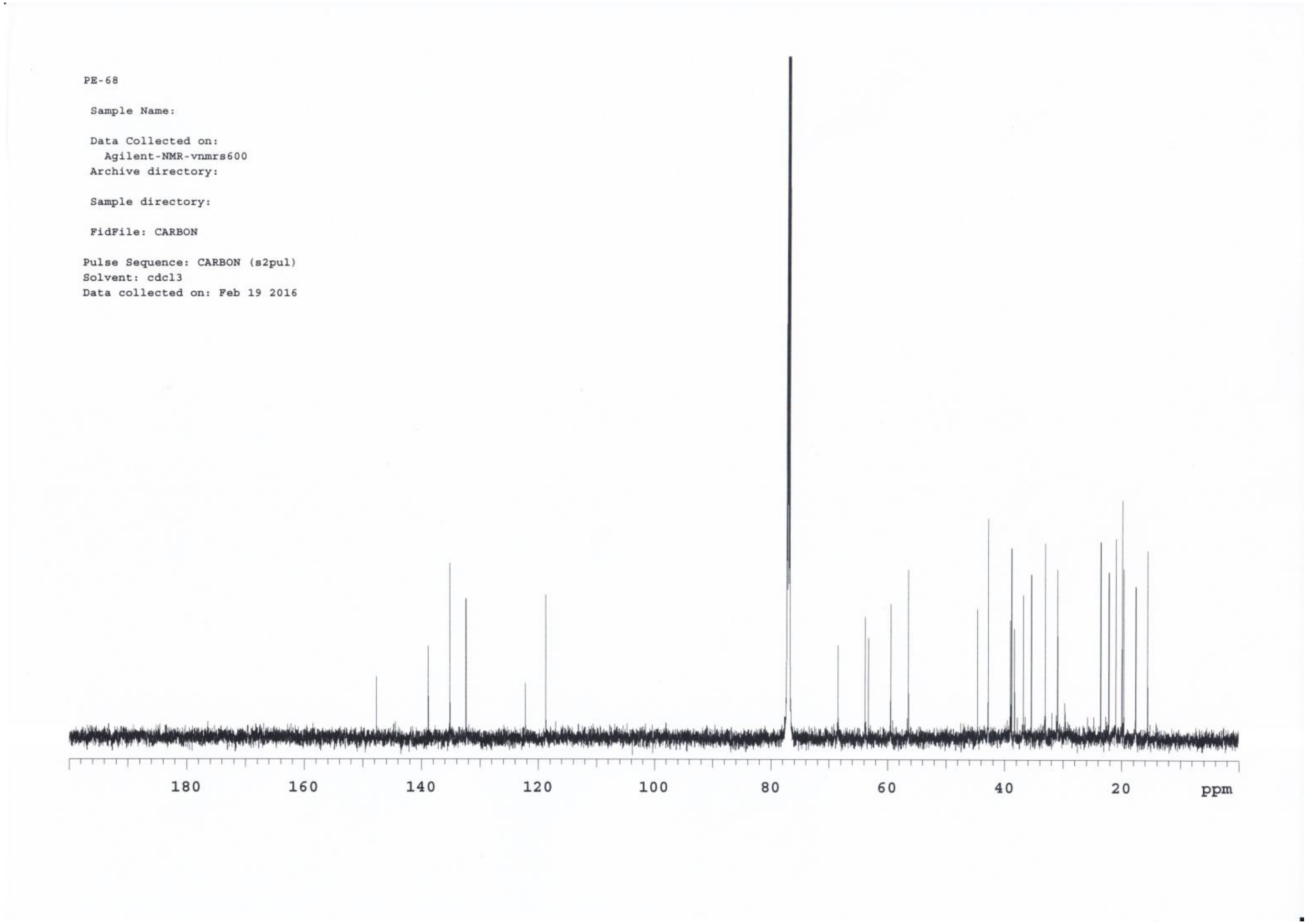

Figure S3. DEPT spectrum of compound **1**.

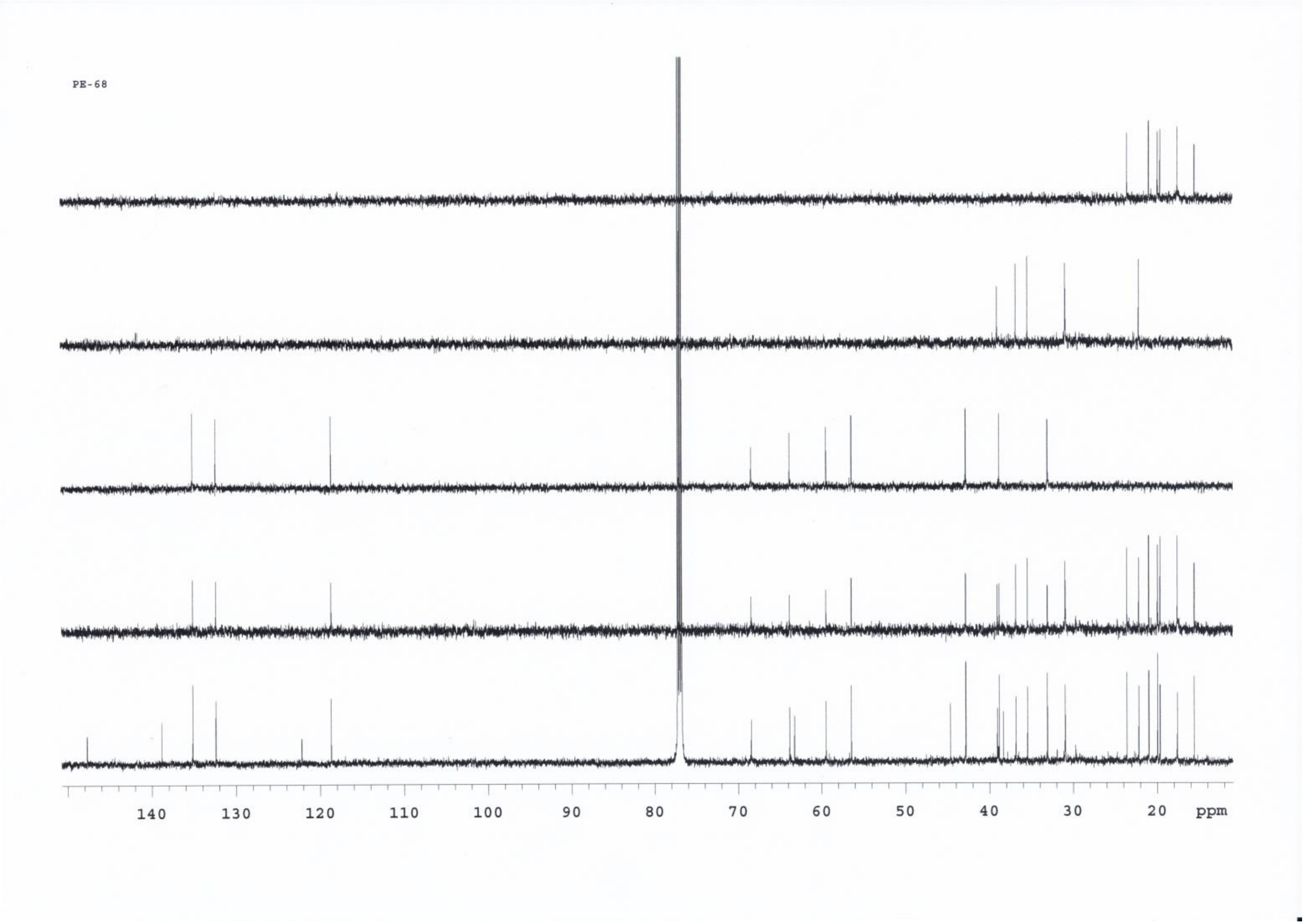

Figure S4. HSQC spectrum of compound **1**.

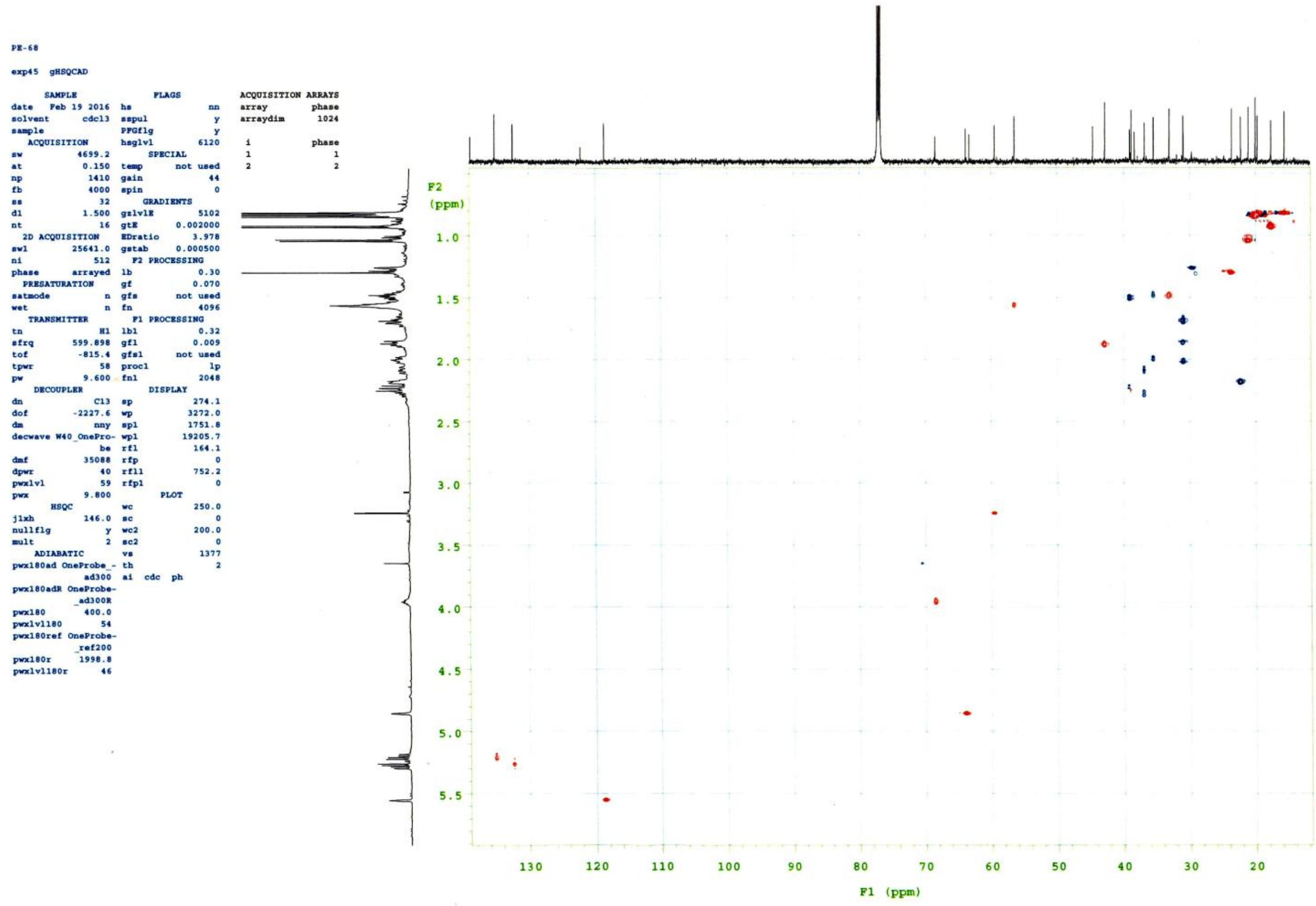

Figure S5. HMBC spectrum of compound **1**.

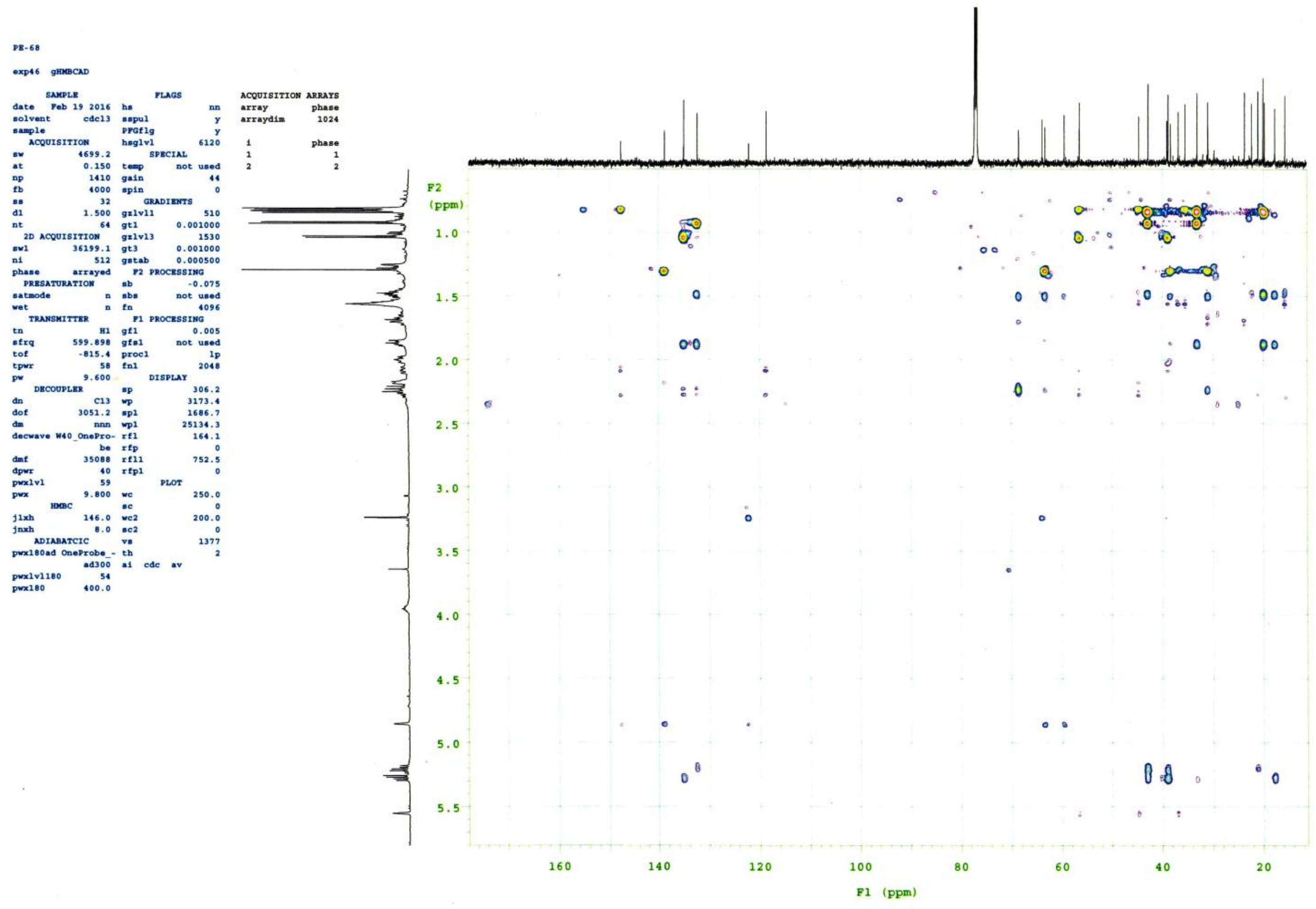

Figure S6. ^{1}H-^{1}H COSY spectrum of compound **1**.

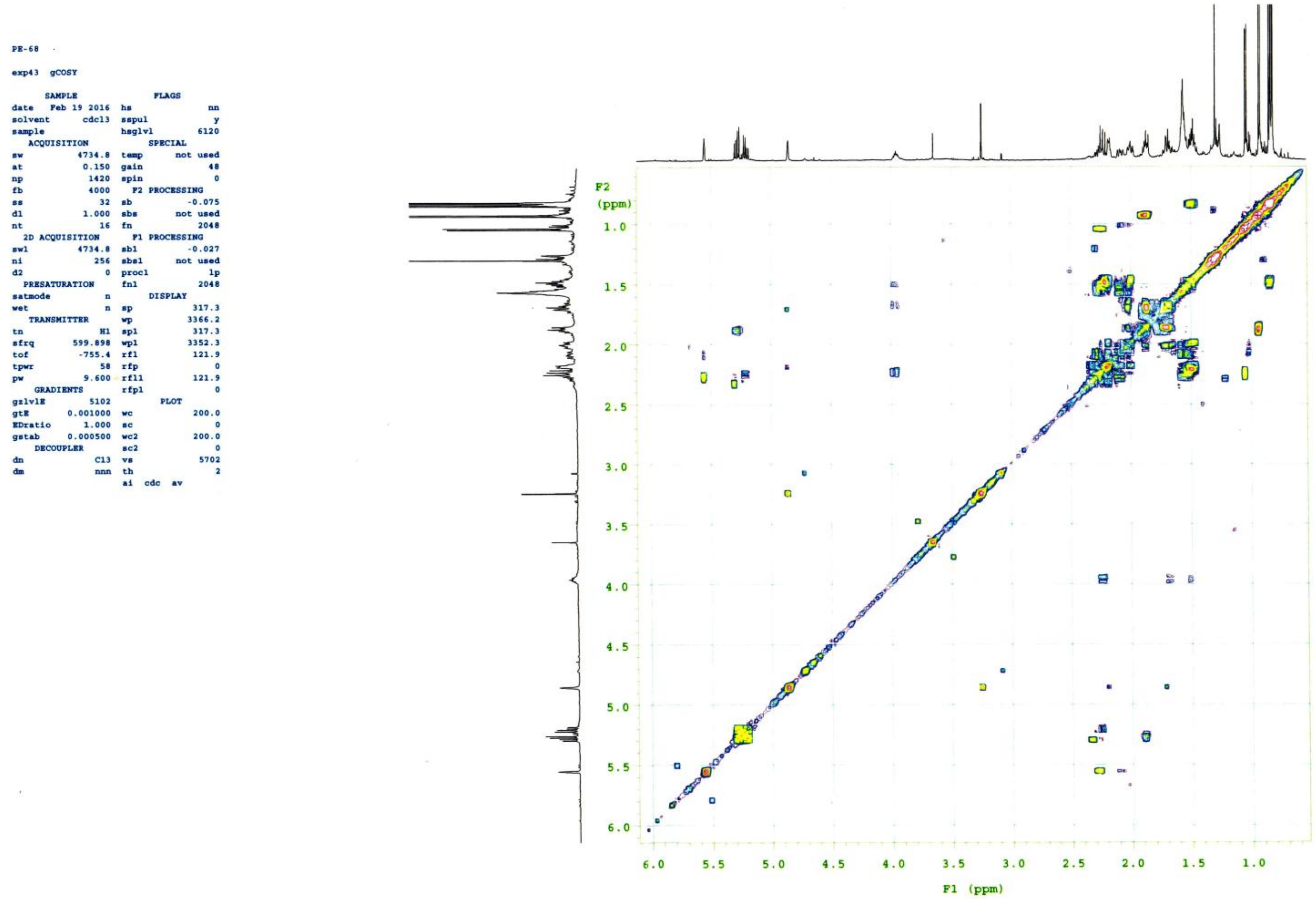

Figure S7. NOESY spectrum of compound **1**.

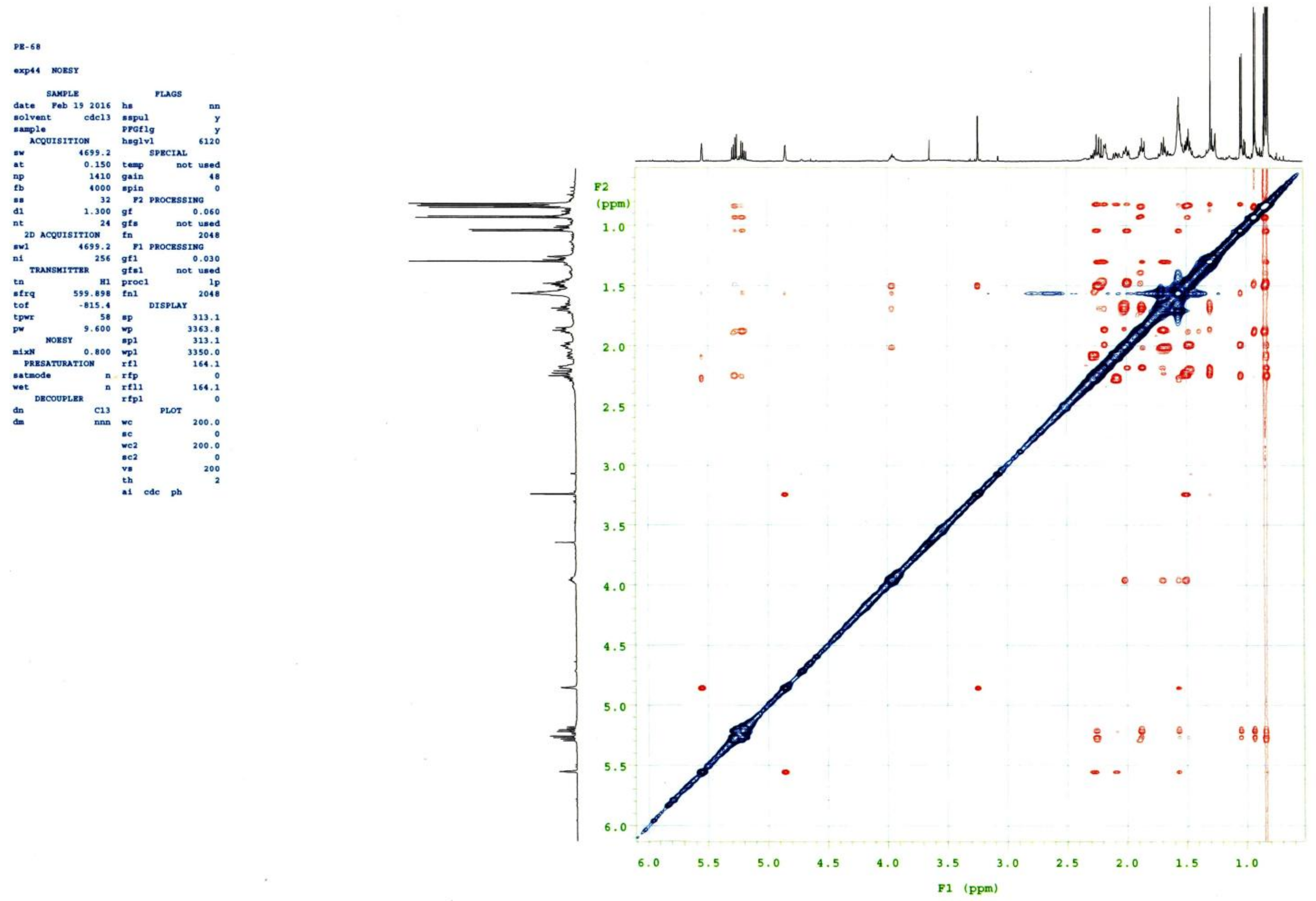

Figure S8. ^{1}H NMR spectrum of compound **2**.

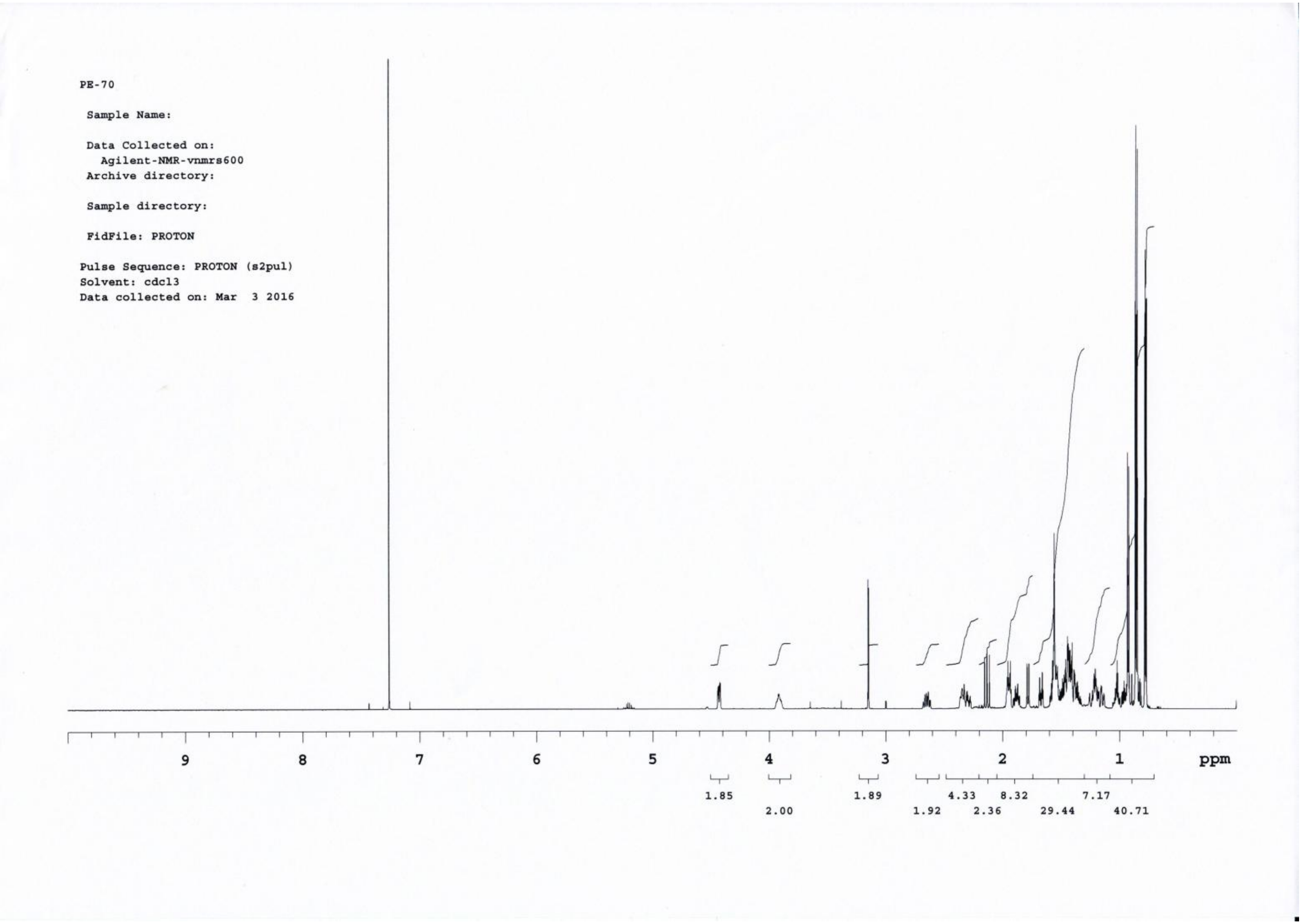

Figure S9. ^{13}C NMR spectrum of compound **2**.

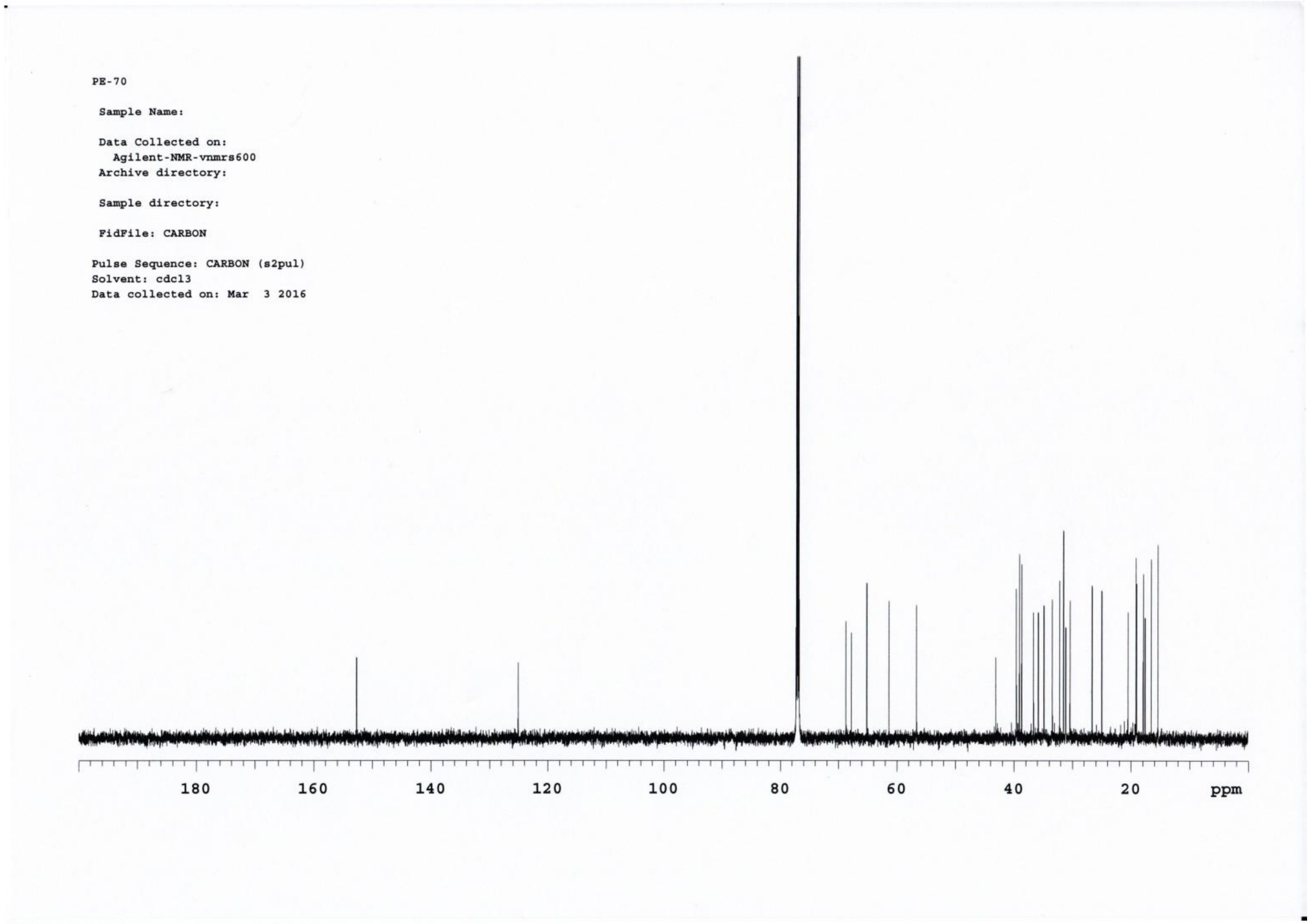

Figure S10. DEPT spectrum of compound **2**.

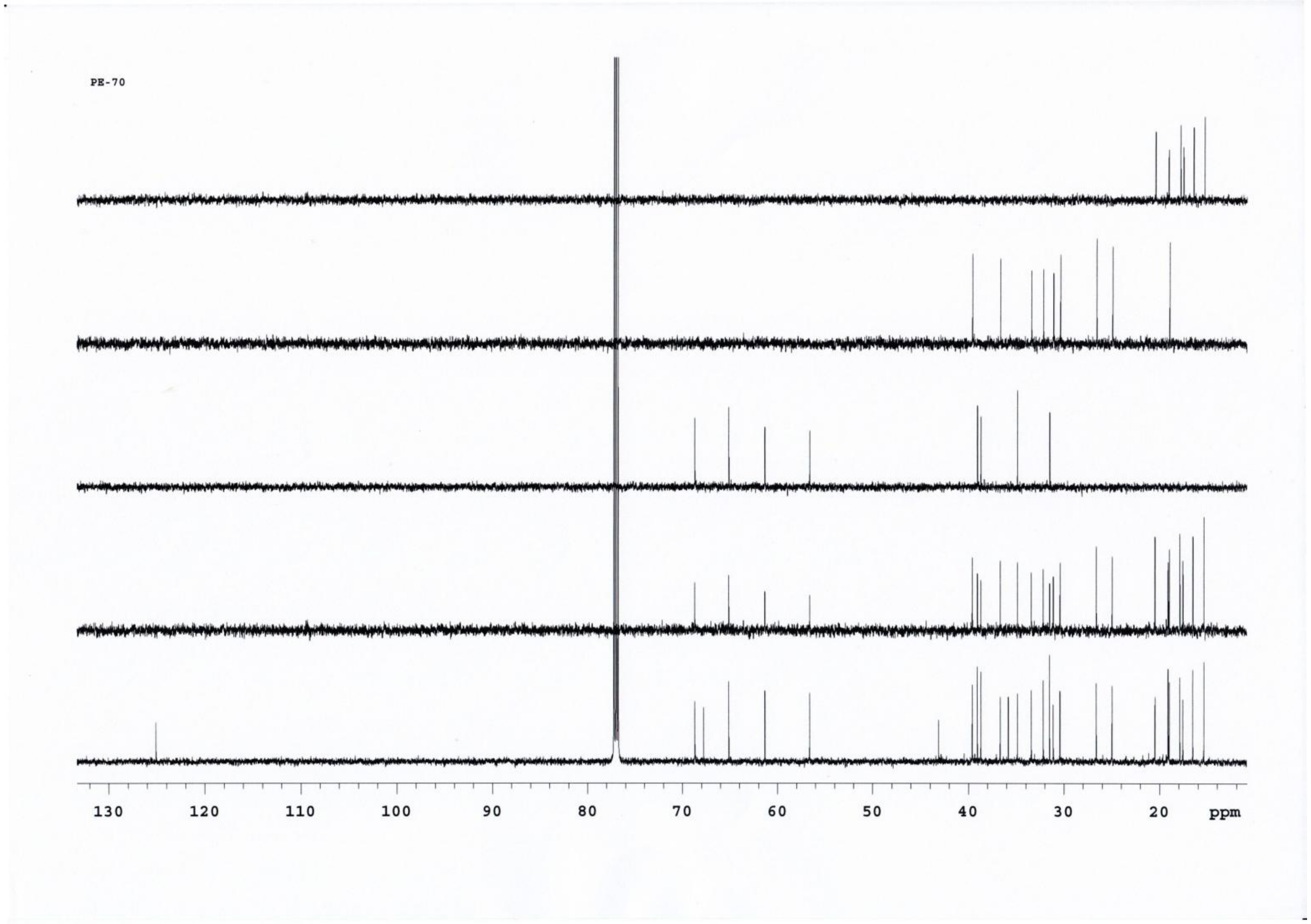

Figure S11. HSQC spectrum of compound **2**.

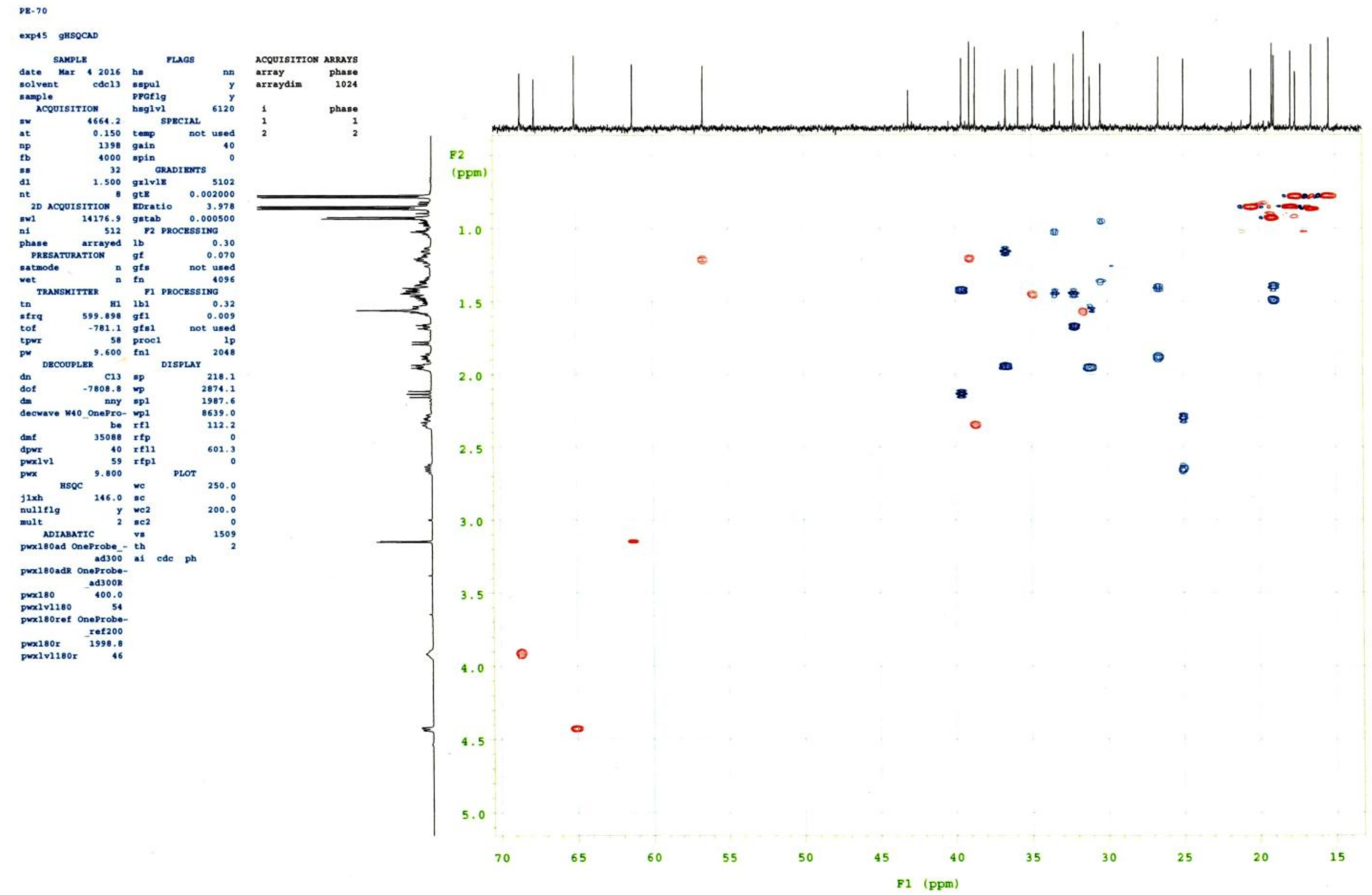

Figure S12. HMBC spectrum of compound **2**.

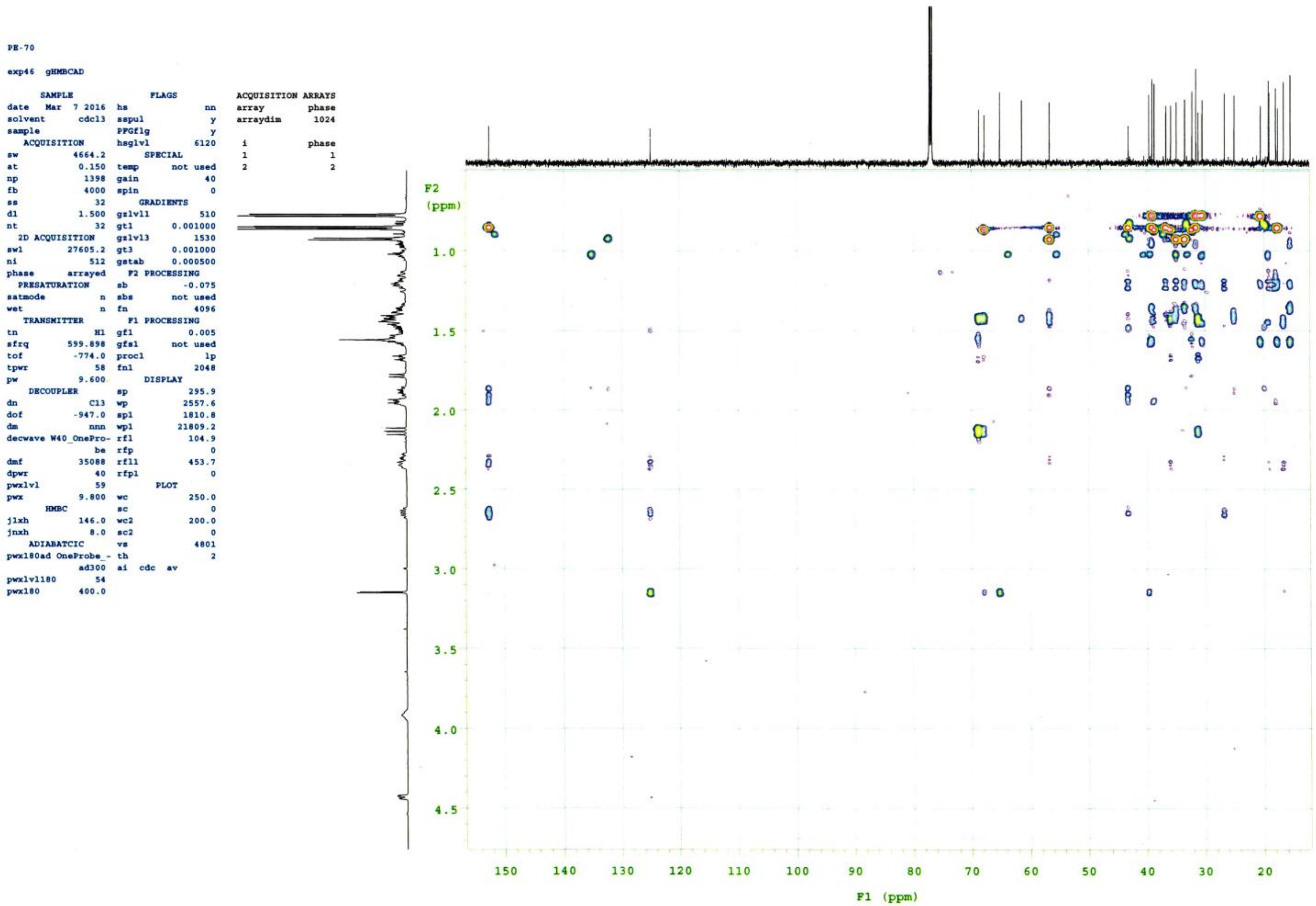

Figure S13. ^{1}H-^{1}H COSY spectrum of compound **2**.

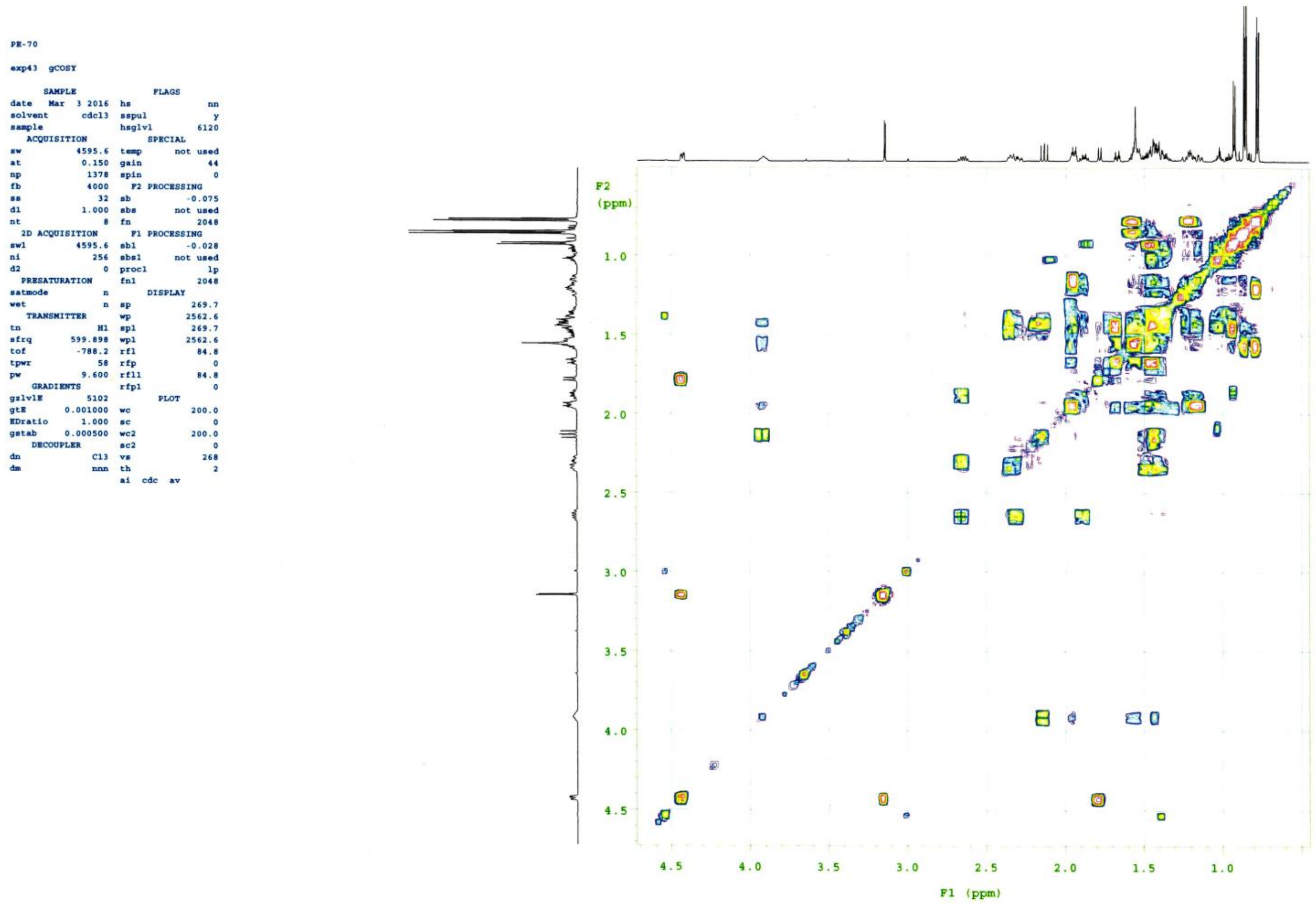

Figure S14. NOESY spectrum of compound **2**.

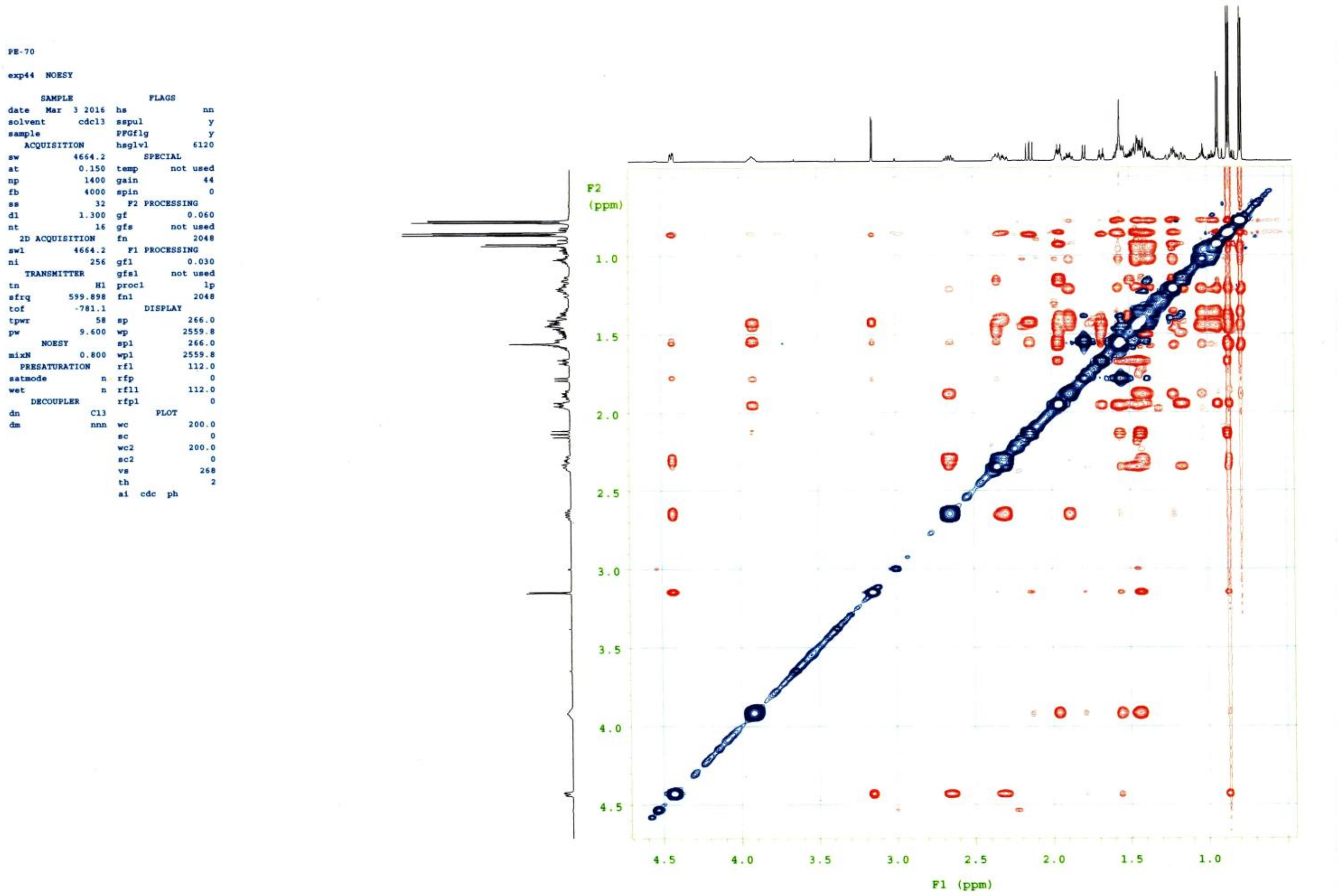

Supplement: Supplementary file 1 [file ijms-18-02479-s001.pdf]
